# Fecal indicator bacteria along multiple environmental exposure pathways (water, food, and soil) and intestinal parasites among children in the rural northwest Ethiopia

**DOI:** 10.1186/s12876-022-02174-4

**Published:** 2022-02-27

**Authors:** Zemichael Gizaw, Alemayehu Worku Yalew, Bikes Destaw Bitew, Jiyoung Lee, Michael Bisesi

**Affiliations:** 1 Department of Environmental and Occupational Health and Safety, Institute of Public Health, College of Medicine and Health Sciences, University of Gondar, Gondar, Ethiopia; 2 Addis Continental Institute of Public Health, Addis Ababa, Ethiopia; 3 Global One Health Initiative (GOHi), The Ohio State University, Columbus, OH USA; 4 School of Public Health, Addis Ababa University, Addis Ababa, Ethiopia; 5 Division of Environmental Health Sciences, College of Public Health, The Ohio State University, 1841 Neil Avenue, Columbus, OH 43210 USA; 6 Department of Food Science and Technology, The Ohio State University, Columbus, OH USA

**Keywords:** *E. coli* contamination of water, *E. coli* contamination of ready-to-eat foods, *E. coli* contamination of courtyard soil, Hand hygiene, Mouthing of soil contaminated materials, Sanitation, East Dembiya district

## Abstract

**Background:**

Children in low-resource settings are exposed to multiple risk factors for enteropathogens. However, the probability of exposures may be different across exposure pathways. Accordingly, this study was conducted to assess environmental exposures of children to intestinal parasites in the east Dembiya district of Ethiopia.

**Methods:**

A cross-sectional study was conducted for 372 households with children aged 24–59 months. The potential for external exposure of children to intestinal parasites was assessed by determining the presence of fecal indicator organism (*Escherichia coli* (*E. coli*)) in drinking water at point of use, ready-to-eat foods, and courtyard soil from children’s outdoor play areas. For internal exposure assessment, ova of parasites in stool samples was detected using wet mount and Kato-Katz techniques to estimate exposure to intestinal parasites. The external and internal exposure assessments were also complemented using questionnaire and spot-check observations to assess behaviors that result in high risk of exposure. Individual and community-level predictors of intestinal parasites were identified using a multilevel logistic regression model. Statistically significant variables were identified on the basis of adjusted odds ratio (AOR) with 95% confidence interval (CI) and *p*-value < 0.05.

**Results:**

Ova of one or more intestinal parasites was detected among 178 (47.8%) (95% CI 42.8, 52.6%) of the children. The most common intestinal parasites were *A. lumbricoides* (20.7%) and *S. mansoni* (19.1%). Furthermore, *E. coli* was detected in 69.1% of drinking water samples at point of use, 67.5% of ready-to-eat food samples, and 83.1% of courtyard soil samples from children’s outdoor play areas. Exposure of children to intestinal parasites among children in the studied region was associated with poor hand hygiene of mothers (AOR 1.98, 95% CI (1.07, 3.66), poor hand hygiene of children (AOR 3.20, 95% CI (1.77, 5.77), mouthing of soil contaminated materials (AOR 2.31, 95% CI (1.26, 4.24), open defecation practices (AOR 2.22, 95% CI (1.20, 4.10), limited access to water (AOR 2.38, 95% CI (1.13, 5.01), water contamination (AOR 2.51, 95% CI (1.31, 4.80), food contamination (AOR 3.21, 95% CI (1.69, 6.09), and soil contamination (AOR 2.56, 95% CI (1.34, 4.90).

**Conclusion:**

An extensive *E. coli* contamination of water, foods, and courtyard soil was found in the studied region and the potential sources of contamination were open defecation practices, unhygienic disposal of wastes, poor animal husbandry and keeping practices, and poor water and food safety measures at household level. Moreover, fecal contamination of water, foods, and soil linked to exposure of children to intestinal parasites in the studied region. Thus, it is critical to implement individual-level interventions (such as latrine utilization, hand hygiene promotion, food safety, home-based water treatment, and containment of domestic animals), plus community-level interventions (such as protecting water sources from contamination, source-based water treatment, and community-driven sanitation).

## Background

Enteric infections are the leading causes of death in children, especially in developing countries. Enteric infections are the cause for approximately 589,000 deaths among children under the age of 5-years in 2017 all over the world [1]. Intestinal parasitic infections are among the most common enteric infections worldwide. More than 1.5 billion people were infected with soil-transmitted helminth infections (STHs) in 2020 [2] and latest estimates indicate that more than 880 million children are in need of treatment for these parasites [3]. Population at risk in African is estimated at 350 million, with the highest infection rate per population occurring in sub-Saharan Africa [3]. About 79 million people are living in STHs endemic areas in Ethiopia, out of which 9.1 million are pre-school-aged children [4].

The burden of enteric infections is high among children compared to the adult population [5]. Children, especially in low-resource settings experience to a variety of enteropathogen risk factors from various sources and exposure pathways (e.g., water, soil, and food). Enteropathogens spread from feces to new hosts via complicated, environmentally mediated pathways. Fecal pathogens can spread into fields and ambient waters in areas with poor sanitation. These are then transported into drinking water and food by fomites (e.g., containers) and vectors (e.g., hands and flies) and ingested by young children through mouth contact with contaminated hands and objects [6].

In areas where children have multiple environmental exposures to enteropathogens, identifying the sources of exposure is critical for planning and implementing effective strategies to reduce transmission of enteric infection [7]. There are numerous approaches for measuring human exposure to enteropathogens along the environmental exposure pathways, ranging from those that measure environmental concentrations of contaminants to predict exposures before the contaminant reaches the human boundary (external exposure assessment) to those that estimate a dose after the contaminant has been taken up into the body (internal exposure assessment) and measurement of host interaction with the environment, as documented by Goddard et al. [7].

The detection of indicators of fecal contamination in environmental samples is a common approach for external exposure assessment. The most common indicator organism of fecal contamination is *E. coli*, which is more specific to warm blooded animals and can be used to track source of environmental contamination [8]. However, measuring environmental exposures using indicator organisms of fecal contamination does not precisely estimate how much enteropathogen crossed the human body envelope [7]. Internal exposure assessments using biological specimens, on the other hand, estimate enteropathogen exposure after it has passed through the human body. Internal exposure assessment, however, provides limited information for tracking the source of environmental contamination [9]. Exposure to enteropathogens is not only conditional on pathogen presence in the environment, but also on host interaction with that environment. Capturing behaviors that result in high risk of exposure using survey or observational data complements exposure assessments and can enable the targeting of environmental media and locations where the study population is predominantly exposed [7]. The SaniPath exposure assessment tool is an important tool to collect environmental samples and information on behaviors that result high risk of exposure. This approach follows the framework for quantitative microbial risk assessment, with an emphasis on hazard identification, exposure assessment, risk characterization, and risk management [10, 11].

Even though, numerous approaches for measuring human exposure along the environmental exposure pathway continuum, are available, evidence is limited in Ethiopia that document the exposure pathways of children to intestinal parasites. Moreover, the probability of exposures may be different across exposure pathways and little is known about which conditions pose the greatest risk for pathogen exposure. Accordingly, this study was conducted to assess environmental exposures of children to intestinal parasites in the east Dembiya district of Ethiopia.

## Methods

### Study settings

This study was conducted in the rural settings of the east Dembiya district of Ethiopia from 01 May to 18 June 2021, which was a dry season. The east Dembiya is one of the districts in central Gondar zone, the Amhara national regional state, Ethiopia. As of July 2020, the district had a total of 192,020 rural and 18,741 urban residents [12], of these, 39,927 (12.22%) were children under age 5-years [13]. In the district, coverage of clean water and latrine were 26.6% and 55%, respectively. Moreover, intestinal parasitic infections were the top four prevalent diseases, which accounted 5161 (9.97%) [14].

### Study design

A community-based cross-sectional study design with structured observation and laboratory investigations was employed to measure environmental exposures of children to intestinal parasites. We used the Goddard et al. [7] and the SaniPath exposure assessment [11] approaches to assess exposure of children to intestinal parasites. These approaches are discussed in more detail in the background section.

### Sample size determination

Sample size was calculated using double population proportion formula with the following assumptions: prevalence of STH among preschool age children who had access to drinking water from protected spring in Chuahit, northwest Ethiopia = 53.3% and prevalence among children who had access to drinking water from well water = 31.6% [15], Z_α/2_ at type 1 error of 5% = 1.96, *Z*_*β*_ at 80% power = 0.842, and allocation ratio = 1:1. Therefore, the sample size n = 81. After considering a design effect of 2 and 15% non-response rate, the final sample size in each group was found to be 186, leading to a total of 372 study subjects.

### Sampling procedures

All households in the rural kebeles (the lowest administrative unit in Ethiopia) in the district were considered for sampling. First, we chose six rural kebeles at random out of 28 kebeles using a simple random sampling technique. We allocated equal number of mothers or care givers-child pairs to each kebele. Finally, 372 households with children aged 24–59 months were included in the study using a systematic random sampling technique. Data collectors began collecting data in households located on the right side of local administrators' offices. Assuming that each rural kebele has an average of 200 households [16, 17], a sampling interval (K = 3) was calculated dividing 200 by the kebele's predetermined sample size (i.e., 62). Following that, a number between one and the sampling interval was chosen at random using the lottery method. After the first random start, every third household with children aged 24–59 months was sampled until the desired sample size for each kebele was reached. The younger one was included in the study for households having two children aged 24–59 months.

### Environmental sample collection and transportation

Drinking water samples at point of use, food samples from ready-to-eat foods, and courtyard soil samples from children’s outdoor play areas were collected aseptically for external exposure assessment. Water, foods, and soil were considered to assess external exposure because these three environmental compartments are the principal transmission pathways along the enteric pathogens exposure pathway continuum [18, 19]. To collect stored water, field workers asked mothers or caregivers to provide a glass of water from their primary drinking water storage container, as if they were giving it to their children, and pour 100 ml into a sterilized sampling bottle [19]. To collect soil samples, the respondents were asked to identify the outdoor area where the youngest child aged 24–59 months had most recently spent time and field workers then scraped the top layer of soil into a sampling bag with a sterile scoop to collect approximately 50 g of soil [19]. To sample ready-to-eat foods, field workers asked mothers or care givers to provide approximately 2 g of food in the same manner they feed their children and we scooped the whole portion to fill a sterile plastic bag (Minigrip GreenLine Biodegradable Reclosable Zipper Bag) using a sterile spoon [19].

All samples were preserved on ice and transported to the laboratory to be processed on the same day, typically within 6 h of collection. Upon arrival at the laboratory, samples were kept on ice until they were processed.

### Stool sample collection

Field workers first explained the purpose of collecting stool from children to mothers or caregivers, and then asked them to tell their children to defecate. To avoid urine contamination of the stool, field workers or mothers or care givers instructed the child to urinate first without pooping. Field workers then handed out paper to mothers or caregivers, instructing them to have their child defecate on it to avoid stool contamination with soil or dirt. Field workers used wooden stick to transfer approximately 50 g of the last part of the stool, the softest part, into the collection container after the child defecated on the paper. The field workers then immediately delivered the sample to the stool examination team, who stationed at the center of the village where stool samples were collected in order to facilitate fresh stool analysis.

### Household data collection

To measure host interactions with the environment, interviewers-administered questionnaire and spot-check observations were used to collect information on behaviors that result in high risk of exposure. Questionnaire and observation checklists were prepared based on a review of relevant literature. The tool was first prepared in English language and translated to the local Amharic language by two native Amharic speakers fluent in English, and back-translated into English by two independent English language experts fluent in Amharic to check consistency. After translation, the tool was tested for validity and internal consistency (CVR: 0.95, I-CVI: 0.97, S-CVI/UA: 0.95, modified kappa: 0.97, and Cronbach’s alpha for internal consistency: 0.85). The tool was organized in to eight parts: (i) socio-demographic information, (ii) access to health and sanitation information, (iii) personal hygiene, (iv) waste management practices, (v) drinking water quality and safety measures, (vi) food hygiene and safety measures,( vii) housing conditions, and (viii) childhood diarrheal disease. Field workers observed child behaviors that would result in hand or mouth contact with environmental fomites (mouthing of soil or soil contaminated materials, such as objects or foods on the ground, eating dirt, mouthing hands, etc.) for 30 min spot observation. Field workers also observed the presence of human or animal excreta in the living environment. Furthermore, handwashing data were gathered by assessing mothers' or caregivers' usual handwashing behavior using self-reports. Field data collectors also looked at the hands of mothers or care givers, including children, to see the general cleanliness and conditions of fingernails. In addition, field data collectors asked mothers or care givers to demonstrate how they wash their hands on a regular basis, which they evaluated using checklists for effective handwashing.

### Detection of *E. coli* in water, food and soil samples

1 g of food and soil samples were homogenized with a sterile peptone-buffered water (PBW, 0.1%) (10 ml for food and 20 ml for soil) using a sterile blending bag and a laboratory-scale processor for 1 min at the specified mixing speed. Serial dilutions were done using sterile distilled water by tenfold dilution. 10 ml of solution from 10^–4^ to 10^–3^ dilutions were taken. The water samples were not diluted before being analyzed. The entire water sample, soil, and food solutions were separately filtered through a 47-mm diameter, 0.45-µm pore-sized sterile filter membrane (Millipore, Burlington, MA, USA) and cultured on membrane lauryl sulphate broth pouring into an absorbent pad (Oxoid Limited, Basingstoke, UK). The prepared samples were incubated for 24 h at 44.5 °C before counting the number of colony forming units (CFU) according to the standard procedures outlined in the WHO guideline [20]. The filtration apparatus was washed with distilled water and flamed between analyses of consecutive samples and sterilized at intervals. The colony number was counted and the results were expressed as CFU per 100 ml of water or 1 g of soil and food samples by taking into consideration of dilution factors. One field blank per sample collectors per week, plus one laboratory blank per laboratory assistants per day were processed for quality control.

Based on the number of colonies of *E. coli* per 100 ml of water sample, the quality of drinking water was taken as conformity (if 0 CFU of *E. coli* per 100 ml), low risk (1–10 CFU/100 ml), intermediate risk (10–100 CU/100 ml), high risk (100–1000 CFU/100 ml), and very high risk (41,000 CFU/100 ml were found) [21]. Furthermore, the quality of ready-to-eat foods was considered "satisfactory" if the mean *E. coli* counts recovered in 1 g of food samples was less than 20 CFU, "borderline" if the mean *E. coli* counts recovered was between 20 and 100 CFU, and "unsatisfactory" if the mean *E. coli* counts recovered was greater than 100 CFU [22].

### Detection of ova of parasites in stool samples

Ova of intestinal parasites in stool samples were detected using direct stool examination (wet mount) and Kato-Katz techniques. Stool specimens were diluted with saline as necessary for direct examination. 0.05 g of stool specimen was placed, mixed with a drop of saline, and covered with a cover slide. Finally, the specimen was examined under the microscope at low (× 10 objective) and high (× 40 objective) magnification powers for the identification of intestinal parasites [23]. A small amount of feces (approximately 2 g) was placed on a scrap piece of paper for the Kato-Katz. Using applicator stick, the stool was pressed against the top of the fecal specimen's screen. The template was placed on a clean microscopic slide and filled with the sieved fecal specimen after the upper surface of the screen was scraped to sieve the fecal specimen. The template was then carefully removed, leaving the entire fecal specimen on the slide. The fecal specimen that remained was covered with a glycerol-soaked cellophane strip and examined under a × 10 objective microscope [23].

The intensity of intestinal parasites was determined based on the number of parasitic eggs per gram (EPG) of the stool sample. The intensity of intestinal parasites was grouped into light (*Ascaris lumbricoides*, 1–4999 EPG; *Hookworm*, 1–1999 EPG; *Schistosoma mansoni,* 1–99 EPG; *Hymenolepis nana*, 1–1999 EPG; *Trichuris trichiura*, 1–999 EPG); moderate (*Ascaris lumbricoides*, 5000–49, 999 EPG; *Hookworm*, 2000–3999 EPG; *Schistosoma mansoni,* 100–399 EPG; *Hymenolepis nana*, 2000–9999 EPG; *Trichuris trichiura*, 1000–9999 EPG); and heavy (*Ascaris lumbricoides*, ≥ 50,000 epg; *Hookworm*, ≥ 4000 EPG; *Schistosoma mansoni,* ≥ 400 EPG; *Hymenolepis nana* and *Trichuris trichiura*, ≥ 10,000 EPG [24, 25].

### Statistical analysis

Stata version 14 (Stata Corp, College Station, TX, USA) was used to analyze data. Multilevel bivariate and multivariable analysis using cluster-level random effects binary logistic regression models were used to assess the independent effects of community factors and moderating effects on the association between individual variables and exposure to intestinal parasites. A two-level binary logistic regression model was applied (i.e., 372 households with children under the age of five-years (level 1) nested within 17 clusters with common water sources (level 2). Both random-intercept and random coefficient logistic models were fitted to estimate associations between the individual and community variables to intestinal parasites using *xtmelogit* Stata command. The null model is fitted without the explanatory variable. The random-intercept logistic models were fitted to assess the influence of unobserved community level characteristics on the prevalence of intestinal parasites allowing the likelihood of exposure to vary randomly across communities assuming the effects of individual characteristics are the same in each community. While the random coefficient model was fitted for drinking water quality at point of use, allowing to vary across communities. Finally, both individual and community variables were adjusted and a cross-level interaction between water sources and water quality at point of use to see evidence of effect modification of the association between water sources and water quality at individual households. We followed a simplified procedures stated by Sommet N and Morselli D [26]. Statistically significant variables were identified on the basis of adjusted odds ratio (AOR) with 95% confidence interval (CI) and *p*-value < 0.05. Intra-cluster correlation (ICC), was calculated to measure the variation between clusters. The fitness of models were assessed using the global Wald’s statistics, the likelihood ratio test of the cluster-level random effects, and Akaike information criterion (AIC).

## Results

### Sociodemographic characteristics of mothers or care givers and children

A total of 372 households with children under the age of five-years participated in this study, with a 100% response rate. The youngest and oldest mothers or care givers ranged from 20 to 45 years, with a mean (± SD) age of 31.7 (± 6.4) years. One hundred twenty-eight (34.4%) of the mothers or care givers were between the ages of 26 and 30 years. The vast majority, 336 (90.3%) of the mothers or care givers were married and 164 (44.4%) of the mothers or care givers can't read and write. The households included in the current study ranged in size from 2 to 8 members, with 133 (35.8%) having more than 5 family members. One hundred and ninety-two (51.6%) of the children in this study were male. Children ranged in age from 24 to 59 months, with a mean (± SD) age of 42 (± 12.5) months. One hundred fifty-eight (42.5%) of the children were aged between 48 and 59 months (Table 1).

**Table 1:** Sociodemographic characteristics of mothers or care givers and children in the rural settings of the east Dembiya district, northwest Ethiopia, May–June 2021, (n = 372)

Sociodemographic variables	Frequency	Percent
Age of mothers or care givers in years		
20–25	74	19.9
26–30	128	34.4
31–35	64	17.2
36–40	82	22.0
41–45	24	6.5
Marital status of mothers or care givers		
Married	336	90.3
Divorced	25	6.7
Widowed	11	3.0
Education status of mothers or care givers		
Can't read and write	165	44.4
Can read and write	34	9.1
Primary education	57	15.3
Secondary education	68	18.3
Certificate/ diploma	48	12.9
Family size		
$\le$ 5	239	64.2
$>$ 5	133	35.8
Sex of child		
Male	192	51.6
Female	180	48.4
Age of children in months		
24–36	92	24.7
37–48	122	32.8
49–59	158	42.5

### Hand hygiene of mothers or care givers and children

In the current study, 362 (97.3%) of the mothers or care givers reported that they always washed hands before feeding, 296 (79.6%) washed before preparing foods, and 276 (74.2%) washed after defecating their children. One hundred fifty-seven (42.2%) and 130 (34.9%) of the mothers or care givers reported they washed hands with water alone and with soap, respectively. Furthermore, 122 (32.8%) and 200 (53.8%) of mothers or care givers reported that they always washed hands of children after they played and before they ate, respectively. Results from the observation revealed that only 65 (17.5%) of the mothers or care givers thoroughly rubbed all parts of their hands for at least 20 s, and 44 (11.8%) of the mothers or care givers wiped their hands on their cloth to dry. Furthermore, 253 (68.0%) of the mothers or care givers and 215 (57.8%) of the children did not keep their fingernails short and clean. Two hundred and forty-seven (66.4%) of the children mouthed soil contaminated materials (Table 2).

**Table 2:** Hand hygiene practice of mothers or care givers and children in the rural settings of the east Dembiya district, northwest Ethiopia, May–June 2021, (n = 372)

Hand hygiene practice	Frequency	Percent
Mothers or care givers always washed hands		
After visiting toilet	266	71.5
After defecating a child	276	74.2
Before feeding a child	362	97.3
Before preparing foods	296	79.6
After handling rubbish	326	87.6
After touching animals	304	81.7
What mothers or care givers usually used to wash hands		
Water alone	157	42.2
Soap	130	34.9
Leaf	17	4.6
Ash	54	14.5
How mothers or care givers washed hands during the observation		
Used soap	118	31.7
Used ash	21	5.6
Thoroughly rubbed all parts of the hand for at least 20 s	65	17.5
Wiped hands on their cloth after washing	44	11.8
Dried in the air after washing	328	88.2
Mothers or care givers always washed hands of children after playing		
Yes	122	32.8
No	250	67.2
Mothers or care givers always washed hands of children before eating		
Yes	200	53.8
No	172	46.2
Mothers or care givers kept fingernails short and clean		
Yes	119	32.0
No	253	68.0
Children kept fingernails short and clean		
Yes	157	42.2
No	215	57.8
Children mouthed soil material		
Yes	247	66.4
No	125	33.6

### Waste management practices

This study revealed that 130 (34.9%) of the rural households used traditional pit latrine 242 (65.1%) of the households defecated in the open field. Three hundred and nineteen (85.8%) of the rural households disposed domestic waste water everywhere in the yard. Two hundred and seventy-six (74.2%) of the rural households disposed of rubbish in the open field. Furthermore, animal excreta in the living environments was observed among 272 (73.1%) the rural households (Table 3).

**Table 3:** Waste management practices of the rural households in the east Dembiya district, northwest Ethiopia, May–June 2021, (n = 372)

Waste management related variables	Frequency	Percent
Defecation practice of household members		
Open field	242	65.1
Traditional pit latrine	130	34.9
How the household manage domestic waste water		
Use soak pit	53	14.2
Disposed everywhere in the yard	319	85.8
How the household manage rubbish		
Open dumping	276	74.2
Burning	79	21.2
Burial	17	4.6
Animal excreta in the living environment		
Yes	272	73.1
No	100	26.9

### Drinking water supply and safety measures

The drinking water source for 283 (76.1%) of the households was ground water. One hundred and ninety-six (52.7%) of the households collected drinking water from unprotected sources (rivers, unprotected springs, or unprotected wells). One hundred forty-two (38.2%) of the households reported that their water sources are intermittent, and 78 (21.0%) of the households reported that they had to travel more than 1 km to access water sources. Vast majority, 299 (80.4%) of the households collected less than 20 l/c/d of water. Two hundred and eighty-four (76.3%) of the households stored water in narrow mouthed containers. At the time of the survey, the water storage containers in 166 (44.6%) and 129 (34.7%) of the households were not clean and covered, respectively. The drinking water in 59 (15.9%) of the households was turbid. Only 14 (3.8%) of the households reported that they are practicing home-based water treatment (Table 4).

**Table 4:** Access to drinking water sources and water handling practices in the rural households in the east Dembiya, northwest Ethiopia, May–June 2021, (n = 372)

Drinking water supply related variables	Frequency	Percent
Drinking water sources		
Ground water	283	76.1
Surface water	89	23.9
Drinking water sources		
Protected	176	47.3
Unprotected	196	52.7
Water sources provide water throughout the year		
Yes	230	61.8
No	142	38.2
How far the water sources located from the dwelling		
Within 1 km radius	294	79.0
More than 1 km away	78	21.0
Volume of water collected		
< 20 l/c/d	299	80.4
$\ge$ 20 l/c/d	73	19.6
Type of water storage containers		
Narrow mouthed containers	284	76.3
Wide mouthed containers	88	23.7
Water storage containers are clean		
Yes	206	55.4
No	166	44.6
The water storage containers are properly covered at the time of the survey		
Yes	243	65.3
No	129	34.7
The water is turbid		
Yes	59	15.9
No	311	84.1
Homebased water treatment		
No	358	96.2
Water guard	6	1.6
Boiling	8	2.2

### Food safety measures

Two hundred and ninety-six (79.6%) of the households reported that they always washed food utensils with soap or ash, and 100 (26.9%) of the households reported that they used a perforating rack to dry washed utensils. Three hundred and thirty-five (90.1%) of the households reported that they thoroughly cooked foods. One hundred and seventy-two (46.2%) and 160 (44.0%) of the mothers or care givers reported that they did not touch their body or other things while preparing food, and did not prepare food while they were experiencing diarrhea or vomiting, respectively. One hundred and fifty-eight (42.5%) of the households reported giving leftover foods to children and 119 of 158 (75.3%) households reheated leftover foods before use. Results from the observation indicated that food utensils containing foods were clean in 225 (60.5%) of the households, covered properly in 233 (62.6%) of the households, stored in clean area or shelf in 199 (53.5%) of the households, were accessible to pets in 118 (31.7%) of the households, and mechanical vectors or rodents were seen around food storage areas in 244 (65.6%) of the households (Table 5).

**Table 5:** Food safety practices of the rural households in the east Dembiya district, northwest Ethiopia, May–June 2021, (n = 372)

Food safety measures	Frequency	Percent
Always washed food utensils with soap or ash		
Yes	296	79.6
No	76	20.4
How do you dry washed food utensils		
Perforating rack	100	26.9
Wipe with cloth	46	12.4
Dry in the air	226	60.8
Thoroughly cooked foods to be cooked		
Yes	335	90.1
No	37	9.9
Does not touch body and other things while preparing foods		
Yes	172	46.2
No	200	53.8
Prepare foods while you have diarrhea/or vomiting or other enteric infections		
Yes	212	57.0
No	160	43.0
Provide leftover foods to children		
Yes	158	42.5
No	214	57.5
Reheat leftover foods before serving (n = 158)		
Yes	119	75.3
No	39	24.7
Food utensils containing foods are clean during the survey		
Yes	225	60.5
No	147	39.5
Food utensils containing foods are covered properly during the survey		
Yes	233	62.6
No	139	37.4
Food utensils containing foods are stored in clean area or shelf during the survey		
Yes	199	53.5
No	173	46.5
Food utensils containing foods are accessible to pets		
Yes	118	31.7
No	254	68.3
Vectors or rodents are seen in food storage areas		
Yes	244	65.6
No	128	34.4

### Detection of *E. coli* in water, food and soil

**Fig. 1:**
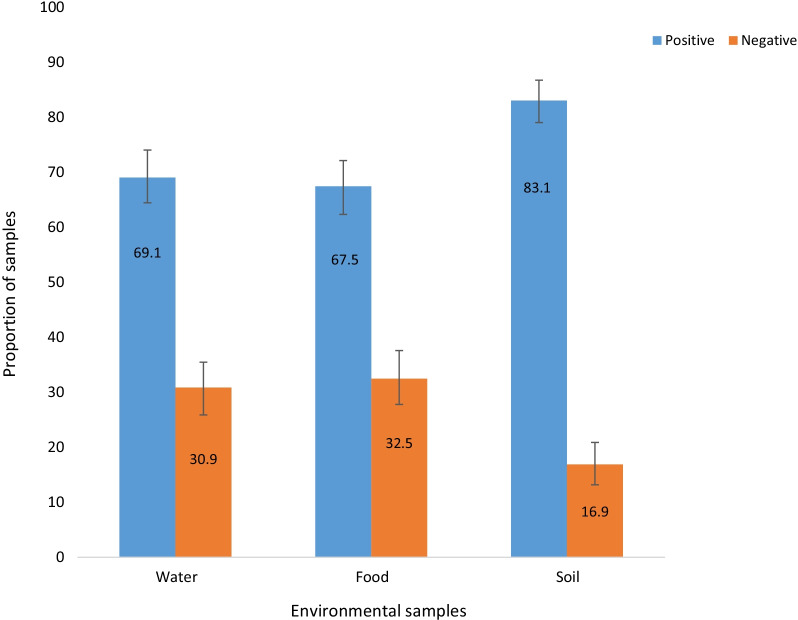
Proportion of positive and negative water, food and soils samples for *E. coli* in the rural households of the east Dembiya district, northwest Ethiopia, May–June 2021, (n = 372). Error bars indicate the 95% CI for proportion

**Fig. 2:**
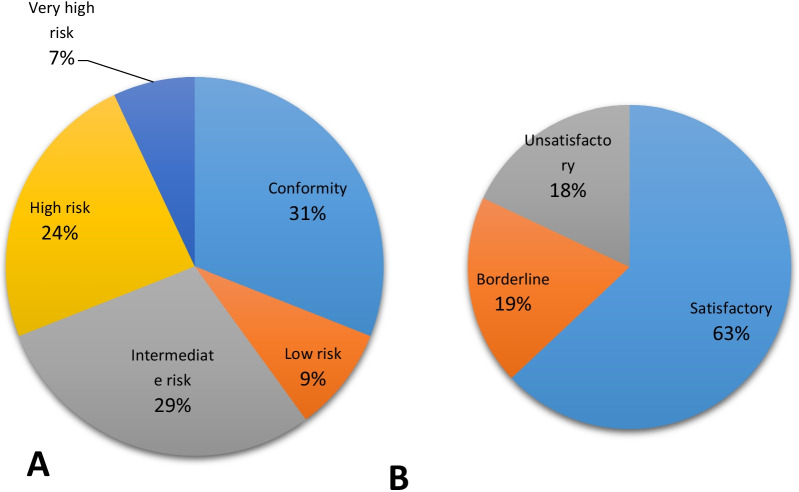
Proportion of households with different risk level of drinking water (**A**) and ready-to-eat foods (**B**) in the rural setting of the east Dembiya district, northwest Ethiopia, May–June 2021, (n = 372)

Figure 1 shows *E. coli* counts recovered in drinking water at point of use, ready-to-eat foods, and courtyard soil. *E. coli* was detected in 257 (69.1%) of the water samples at point of use. The lowest and highest *E. coli* counts recovered were 1 and 1613 CFU per 100 ml, respectively with a mean *E. coli* count of 273.37 CFU. The water quality of 90 (24%) and 27 (7%) of the households was at high and very high-risk level, respectively (Fig. 2A). Similarly, *E. coli* was detected in 251 (67.5%) of the food samples. The lowest and highest *E. coli* counts recovered were 1 and 1500 CFU per g, respectively with a mean *E. coli* count of 184.88 CFU. The microbial quality of ready-to-eat foods was, therefore, found to be unsatisfactory in 68 (18.3%) of the rural households (Fig. 2B). Three hundred and nine (83.1%) of the soil samples were found to be positive for *E. coli*. The lowest and highest *E. coli* counts recovered were 20 and 3200 CFU per g, respectively with a mean *E. coli* count of 739.18 CFU.

### *E. coli* contamination pathways in soil, water, and food

**Fig. 3:**
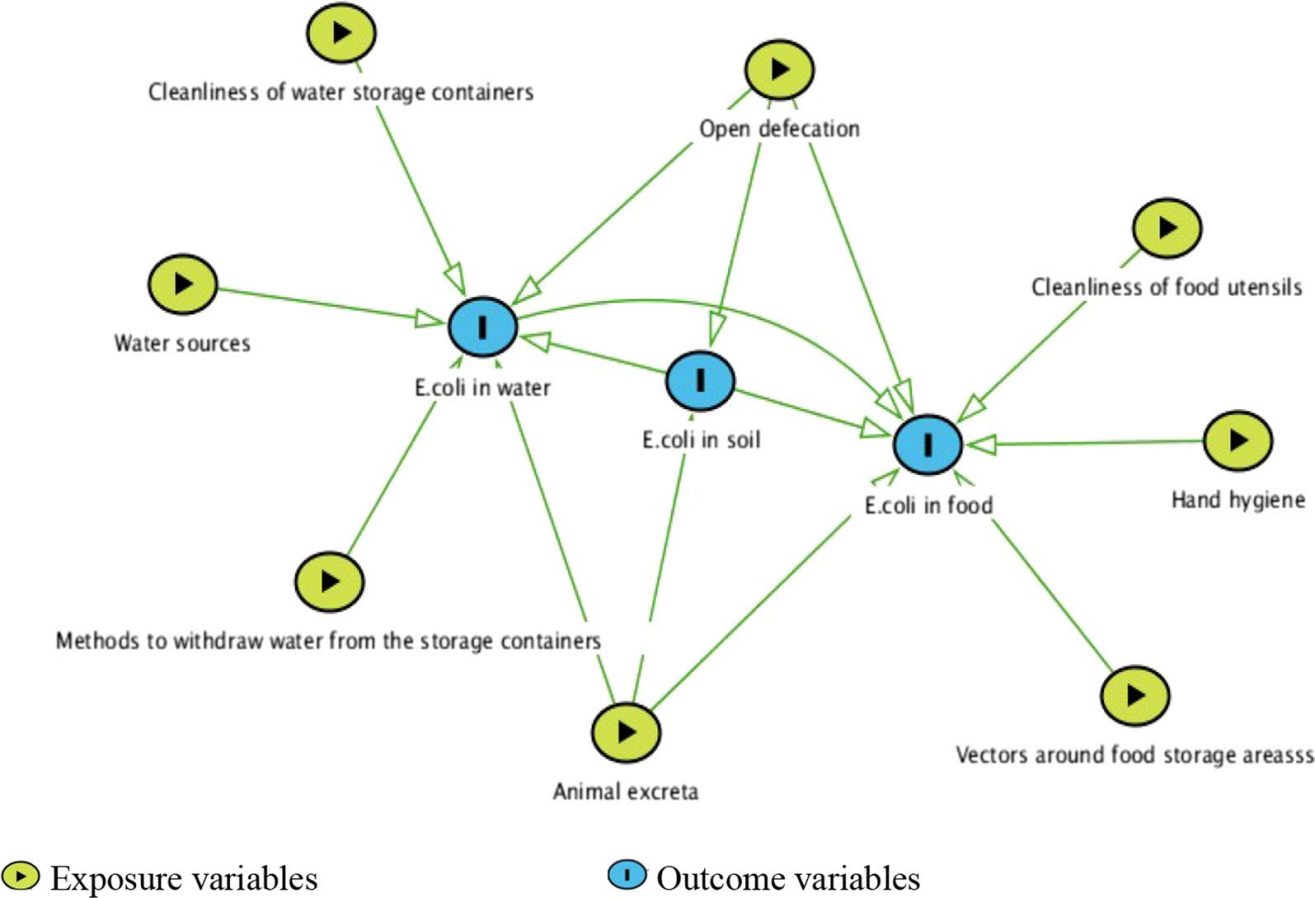
Exposure pathways of *E. coli* contamination along the environmental compartments. The chart is drawn using Dagitty software from the results of a linear regression analysis done to see the associations between contamination levels of environmental exposure pathways along another pathways. Arrows indicate associations that are significant (*p* < 0.05); the lack of an arrow between two sample types indicates that we did not observe a significant association

Figure 3 illustrates the pathways of *E. coli* contamination along the three environmental compartments (water, food, and soil). Number of *E. coli* counts in CFU recovered from courtyard soil in child playing areas was associated with open defecation practice (β: 188.6, 95% CI (64.0, 313.1) and presence of animal excreta in the living environment (β: 592.6, 95% CI (470.7, 714.4). *E. coli* counts recovered in drinking water was explained by *E. coli* contamination of soil (β: 227.4, 95% CI (132.6, 322.1), open defecation practice (β: 127.8, 95% CI (53.6, 202.0), presence of animal excreta in the living environment (β: 126.4, 95% CI (43.8, 209.1), unprotected water sources (β: 19.2, 95% CI (32.5, 205.8), unclean water storage containers (β: 100.0, 95% CI (27.1, 172.9), and dipping of mugs to withdraw water from the storage containers (β: 187.0, 95% CI (90.7, 283.4). *E. coli* counts recovered in ready-to-eat foods was also associated with open defecation practice (β: 71.5, 95% CI (1.6, 141.4), presence of animal excreta in the living environment (β: 94.2, 95% CI (19.9, 168.5), availability of mechanical vectors around food storage areas (β: 108.6, 95% CI (43.1, 174.2), unclean food utensils (β: 108.8, 95% CI (43.7, 173.8), poor hand hygiene of persons who prepared or served foods (β: 104.4, 95% CI (37.8, 170.9), *E. coli* contamination of soil (β: 91.5, 95% CI (4.6, 178.4), and *E. coli* contamination of water (β: 76.7, 95% CI (6.2, 147.2).

### Intestinal parasites in children

**Fig. 4:**
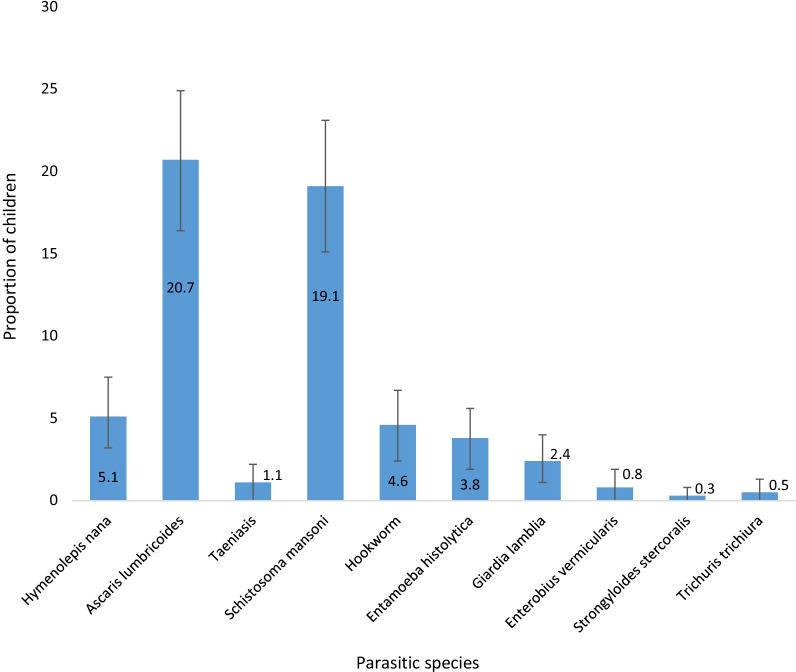
Common intestinal parasites identified among children aged 24–59 months in the rural settings of the east Dembiya district, northwest Ethiopia, May–June 2021, (n = 372). Error bars indicate the 95% CI for proportion of parasitic species

A total of 372 children were examined, with 178 (47.8%) (95% CI: 42.8, 52.6%) of them had ova of one or more intestinal parasites. The commonest intestinal parasites identified among children were *A. lumbricoides* [77 (20.7%)], *S. mansoni* [71 (19.1%)], *H. nana* [19 (5.1%)], *Hookworm* [17 (4.6%)], and *E. histolytica* [14 (3.8%) (Fig. 4). The intensity of *S. mansoni* was moderate and heavy among 25 (6.7%) and 5 (1.3%) of the children, respectively (Table 6).

**Table 6:** Mean number of eggs per gram of stool quantified with minimum and maximum number and intensity of intestinal parasites in children aged 24–59 months in the rural settings of the east Dembiya district, northwest Ethiopia, May–June 2021, (n = 372)

Parasitic species	Mean number of eggs per gram of stool (minimum and maximum)	Number of children with		
		Light intensity	Moderate intensity	Heavy intensity
*A. lumbricoides*	1725 (48, 12,560)	70	7	0
*Hookworm*	95 (48, 384)	17	0	0
*S. mansoni*	217 (24, 3456)	41	25	5
*H. nana*	489 (48, 6480)	18	1	0
*T. trichiura*	252 (120, 384)	2	0	0

### Environmental predictors of intestinal parasites in children

The multilevel (intercept-only) model represents a significant improvement in fit relative to a standard binary logistic regression. The chi-square test indicates the difference in fit is significant between models, χ^2^ (1) = 58.31, *p* < 0.001. The intra-class correlation coefficient (ICC) also shows that there was substantial clustering effect on exposure of children to intestinal parasites. Table 7 illustrates that the community variance in intestinal parasites in children was estimated at 38%. It means that 38% of the residual variation in the exposure of children to intestinal parasites is attributable to unobserved community characteristics.

**Table 7:** Multilevel binary logistic regression results on the environmental predictors of exposure of children to intestinal parasites in the rural settings of the east Dembiya district, northwest Ethiopia, May 2021

Variables	Null model	Intermediate model	Coefficient model	Cross-level interaction model
Age of children in months				
24–36 months		1.0	1.0	1.0
37–48 months		2.16 (1.02, 4.58)*	2.18 (1.01, 4.68)*	2.15 (1.01, 4.58)*
49–59 months		1.46 (0.69, 3.06)	1.63 (0.76, 3.49)	1.45 (0.69, 3.05)
Mothers or care givers kept finger nails short and clean				
Yes		1.0	1.0	1.0
No		1.93 (1.04, 3.57)*	1.98 (1.07, 3.66)*	1.99 (1.07, 3.69)*
Children kept finger nails short and clean				
Yes		1.0	1.0	1.0
No		3.15 (1.75, 5.69)***	3.20 (1.77, 5.77)***	3.24 (1.79, 5.85)***
Mouthing of soil contaminated materials				
Yes		2.21 (1.21, 4.02)**	2.31 (1.26, 4.24)**	2.30 (1.26, 4.18)**
No		1.0	1.0	1.0
Defecation practice of households				
Open field		2.18 (1.18, 4.01)*	2.22 (1.20, 4.10)*	2.31 (1.24, 4.30)**
Latrine		1.0	1.0	1.0
Animal excreta in the living environment				
Yes		1.52 (0.81, 2.83)	1.64 (0.87, 3.11)	1.54 (0.82, 2.88)
No		1.0	1.0	1.0
Volume of water a family collected per day				
< 20 l/c/d		2.47 (1.18, 5.18)*	2.38 (1.13, 5.01)*	2.52 (1.21, 5.26)*
$\ge$ 20 l/c/d		1.0	1.0	1.0
*E. coli* detected in courtyard soil samples				
Yes		2.52 (1.31, 483)**	2.56 (1.34, 4.90)**	2.48 (1.29, 4.75)**
No		1.0	1.0	
*E. coli* detected in ready-to-eat food samples				
Yes		3.09 (1.65, 5.79)***	3.21(1.69, 6.09)***	3.17 (1.68, 5.98)***
No		1.0	1.0	1.0
*E. coli* detected in drinking water samples				
Yes		2.51 (1.31, 4.80)**	1.88 (0.68, 5.22)	5.30 (1.89, 14.85)**
No		1.0	1.0	1.0
Vectors observed in the food storage areas				
Yes		1.09 (0.60, 1.98)	1.12 (0.62, 2.03)	1.14 (0.62, 2.07)
No		1.0	1.0	1.0
Drinking water sources (2nd level predictor)				
Protected		1.0	1.0	1.0
Unprotected		1.81 (0.37, 8.82)	0.70 (0.22, 2.19)	1.26 (0.26, 5.97)
Drinking water source*E. coli detected in water samples at point of use				
Protected*No				1.0
Unprotected*Yes				3.83 (1.01, 14.46)*
Random effects				
Coefficient variance (SE)			0.15 (0.65)	
Community level variance (SE)	2.03 (1.05)	2.35 (1.30)	2.14 (2.24)	2.13 (1.20)
Covariance (SE)			− 0.56 (1.51)	
Log-likelihood	58.31 (*p* < 0.001)	33.36 (*p* < 0.001)	37.81 (*p* < 0.001)	29.33 (*p* < 0.001)
ICC	0.38	0.42	0.04	0.39
AIC	460.71	392.46	392	390.38

Note: * statistically significant variables at *p* < 0.05, ** statistically significant variables at *p* < 0.01, and *** statistically significant variables at *p* < 0.001

The coefficient model shows that exposure to intestinal parasites was higher among older children compared to young children (AOR 2.18, 95% CI (1.01, 4.68). The odds of having intestinal parasites was 1.98 times higher in children whose mothers did not keep their fingernails short and clean (AOR 1.98, 95% CI (1.07, 3.66). Children who didn’t keep their fingernails short and clean had also higher odds to have intestinal parasites compared with their counterparts (AOR 3.20, 95% CI (1.77, 5.77). In addition, the odds of having intestinal parasites was 2.31times higher in children who mouthed soil contaminated materials (AOR 2.31, 95% CI (1.26, 4.24) (Table 7).

Furthermore, children who lived in families who practiced open defecation had higher exposure to intestinal parasites compared with children who lived in families who used latrine (AOR 2.22, 95% CI (1.20, 4.10). The odds of having intestinal parasites was also 2.38 times higher among children who lived in families with no basic access to drinking water (AOR 2.38, 95% CI (1.13, 5.01) and poor water quality was significantly associated with exposure to intestinal parasites (AOR 2.51, 95% CI (1.31, 4.80). The bacteriological quality of ready-to-eat foods was also significantly associated with high burden of intestinal parasites in children (AOR 3.21, 95% CI (1.69, 6.09). Similarly, poor bacteriological quality of courtyard soil was associated with increased probability of exposure of children to intestinal parasites (AOR 2.56, 95% CI (1.34, 4.90) (Table 7).

The cross-level interaction model shows that there is evidence of effect modification of the association between bacteriological quality of drinking water at point of use and exposure of children to intestinal parasites by water sources. For children in households whose drinking water quality is poor, having unprotected water sources (versus protected sources) results in a 3.83 times higher chance of having intestinal parasites (AOR 3.83, 95% CI (1.01, 14.46) (Table 7).

## Discussion

This community-based cross-sectional study with structured observation and laboratory investigations was conducted in the rural settings of northwest Ethiopia to assess environmental exposures of children to intestinal parasites. An extensive *E. coli* contamination of environmental compartments, including drinking water, ready-to-eat foods and courtyard soil from children’s outdoor play area was observed. Although the WHO standard for drinking water quality is 0 CFU of *E. coli* per 100 ml of drinking water [20], we found that the *E. coli* counts in 69.1% of the households exceeded the WHO standard with a mean *E. coli* count of 273.37 CFU per 100 ml. Other studies also reported high *E. coli* contamination of water at household level [27–31]. This high *E.coli* contamination of water can be explained by poor sanitation condition in the area and poor water handling practices at individual households. As we documented in this study, open defecation and animal excreta were common in the study area, which could cause contamination of drinking water at the source. Pathogens in the contaminated environment or soil can reach to water sources by the help of flood, wind, and animals [32–35]. Moreover, types and cleanliness of water storage containers, methods of withdrawing water from the storage containers, and sanitation condition of the storage areas are risk factors for contamination of water at household level [36–38]. As documented in this study, water storage containers in 44.6% of households were not clean and not properly covered in 34.7% of the households, and 96.2% of the households did not practiced home-based water treatment.

In the current study, 83.1% of the courtyard soil samples from child playing areas were positive for *E. coli* with a mean *E. coli* count of 739.18 CFU per g. Other studies in developing countries also reported high *E. coli* contamination of courtyard soil [39–42]. Two major factors explained the high *E. coli* contamination: presence of animal excreta in the living environment and open defecation. Studies done in low and middle income countries also suspected animal excreta as a contributor to fecal contamination [43–46]. In the study area domestic animals and their feces are not properly contained or separated from domestic environments. Domestic animals generate 85% of the world’s animal fecal waste, a far greater proportion than the human population, resulting in fecal contamination of soil due to insufficient separation of animal feces from human domestic environments [47]. Despite the fact that adult family members may not defecate openly in the courtyard, child feces was observed in a significant proportion of rural households, which could lead *E. coli* contamination of courtyard soil. Furthermore, because two-thirds of the households in the study area defecated openly, i.e., near bushes in the living environment, feces could reach the courtyard soil via animals, wind, and flood, resulting *E. coli* contamination of the courtyard soil. Even if a single infected animal may excrete more pathogens than a single human [47–49], 1 g of fresh feces from an infected person can contain around 10^6^ viral pathogens, 10^6^–10^8^ bacterial pathogens, 10^4^ protozoan cysts, and10–10^4^ helminth eggs [50].

*E. coli* contamination of ready-to-eat foods was reported in 67.5% of the food samples collected from individual households with a mean *E. coli* count of 184.88 CFU/g, which indicated that the food in more than two-third of the households was contaminated. Other similar studies also reported high *E. coli* contamination of foods like our study [42, 51–54]. The *E. coli* counts recovered in 37% of the rural households were higher than the microbiological standards of ready-to-eat foods [22, 55]. Findings of the current study suggest that open defecation practice, presence of animal excreta in the living environment, availability of vectors in/around food storage areas, unclean food utensils, and poor hand hygiene of persons who prepared or served foods, *E. coli* contamination of soil, and *E. coli* contamination of water all significantly contribute to *E. coli* contamination of ready-to-eat foods. Foods may not be directly exposed to primary contaminants, i.e., fecal matters, rather fecal matters make their way into foods because of poor personal hygiene of food handlers, contaminated water, and vectors like flies and rodents. This exposure pathways are widely documented in literature [54, 56–58].

The extensive contamination of the home environment (drinking water, ready-to-eat foods and courtyard soil) was strongly associated with exposure of children to intestinal parasites. Ova of one or more intestinal parasites was detected in 47.8% (95% CI 42.8, 52.6%) of the children, which is high compared with reports of community-based studies among under-five children in different parts of Ethiopia, such as in Chuahit (35.2%) [15], Goncha Siso Enese (11.8%) [59], Wonji Shoa (24.3%) [60], and Butajira (23.3%) [61]. Moreover, the burden of intestinal parasites reported in the current study is higher compared to findings of studies in other developing countries, such as in Kenya (25.6%) [62], India (17.0%) [63], Mozambique (31.6%) [64], and Nigeria (23.3%) [65]. The high burden of intestinal parasites in children is linked to extensive *E. coli* contamination water, ready-to-eat foods and courtyard soil from children’s outdoor play area. Fecal contamination of foods, water and soil plays a greater role in fecal–oral transmission [66].

This study also revealed that the high burden of intestinal parasites in children in the rural northwest Ethiopia was significantly associated with open defecation practice and poor hand hygiene condition of mothers or care givers and children and mouthing of soil contaminated materials. Open defecation causes fecal contamination of soil. Cross-contamination of water and foods occurs as a result of fecally contaminated soil [18, 67–69]. In addition, fecally contaminated soil creates favorable conditions for multiplication of mechanical vectors that carry pathogens from contaminated soil to food [70–72]. Contaminated soil also causes contamination of hands with fecal matter while playing or doing day-to-day activities [73, 74]. Children get sick from contaminated hands if they don't wash hands frequently with soap and keep their fingernails short. The area beneath the fingernails has the highest concentration of microorganisms on the hands and is the most difficult to clean [75–78]. Mouthing of soil contaminated materials is also the other direct impact of fecally contaminated soil in exposure to intestinal parasites. Children may ingest diseases causing pathogens when they mouthed soil contaminated materials [41, 79–81]. According to findings of this study, children who mouthed soil contaminated materials had a higher risk of exposure to intestinal parasites. Furthermore, lack of access to water for personal hygiene in the area increases the risk of exposure to intestinal parasites through contaminated hands from fecally contaminated soil [82, 83]. This study discovered that children who lived in families with no basic access to drinking water, i.e., less than 20 l/c/d, had a higher risk of exposure to intestinal parasite.

As a limitation, the burden of intestinal parasites was measured in the dry season. The associations reported in the current study are not adjusted for seasonal variations. We, therefore, recommend other large scale study to clearly show the seasonal variations. Moreover, there might be errors in quantification of ova of parasites because of variation in ova distribution in stool samples. To minimize this error, the preparation was primarily smeared evenly based on the standard to increase the quality of detection and quantification. The self-reported data may not be reliable since the study subjects may make the more socially acceptable answer rather than being truthful and they may not be able to assess themselves accurately. The generalizability of the results may be affected since contamination may vary in different settings.

## Conclusion

An extensive *E. coli* contamination of water, foods, and courtyard soil was found in the studied region and the potential sources of contamination were open defecation practices, unhygienic disposal of wastes, poor animal husbandry and keeping practices, and poor water and food safety measures at household level. Moreover, fecal contamination of water, foods, and soil linked to a high burden of intestinal parasites among children in the studied region, which indicates children in the rural northwest Ethiopia had multiple exposure pathways to intestinal parasites. Administration of anthelminthic drugs to the infected children is important to expel parasitic worms and other internal parasites from the body. Moreover, implementing both individual-level intervention (such as construction and utilization of latrine, safe disposal of wastes, food safety, hand hygiene promotion, household water treatment, and containment of domestic animals and their excreta) and community-level interventions (such as protecting water sources from fecal contamination, source-based water treatment, community-driven sanitation, and WASH behavior change communication) is important to protect the environment from contamination and to prevent child exposure to intestinal parasites and transmission. In general, integrating preventive chemotherapy with WASH interventions to have the greatest impact towards prevention and control of intestinal parasites in the area should be the priority of the local healthcare system as reinfections can be rapid due to the complex nature of exposures.

## Data Availability

Data will be made available upon requesting the primary author.

## Abbreviations

AIC: Akaike information criterion
CFU: Colony forming units
CI: Confidence interval
CVR: Content validity ratio
*E.** coli*: *Escherichia coli*
EPG: Eggs per gram
DAG: Direct acyclic graph
ICC: Intraclass correlation coefficient
I-CVI: Item-level content validity index
l/c/d: Liter per capita per day
ml: Milliliter
PBW: Peptone-buffered water
S-CVI/UA: Universal agreement scale -level content validity index
SD: Standard deviation
STHs: Soil-transmitted helminth infections
WASH: Water, sanitation and hygiene
WHO: World health organization
